# Topological distortion and reorganized modular structure of gut microbial co-occurrence networks in inflammatory bowel disease

**DOI:** 10.1038/srep26087

**Published:** 2016-05-18

**Authors:** Steven N. Baldassano, Danielle S. Bassett

**Affiliations:** 1 Department of Bioengineering, University of Pennsylvania, Philadelphia, PA 19104, USA; 2 Center for Neuroengineering and Therapeutics, University of Pennsylvania, Philadelphia, PA 19104, USA; 3 Department of Electrical & Systems Engineering, University of Pennsylvania, Philadelphia, PA 19104, USA.

## Abstract

The gut microbiome plays a key role in human health, and alterations of the normal gut flora are associated with a variety of distinct disease states. Yet, the natural dependencies between microbes in healthy and diseased individuals remain far from understood. Here we use a network-based approach to characterize microbial co-occurrence in individuals with inflammatory bowel disease (IBD) and healthy (non-IBD control) individuals. We find that microbial networks in patients with IBD differ in both global structure and local connectivity patterns. While a “core” microbiome is preserved, network topology of other densely interconnected microbe modules is distorted, with potent inflammation-mediating organisms assuming roles as integrative and highly connected inter-modular hubs. We show that while both networks display a rich-club organization, in which a small set of microbes commonly co-occur, the healthy network is more easily disrupted by elimination of a small number of key species. Further investigation of network alterations in disease might offer mechanistic insights into the specific pathogens responsible for microbiome-mediated inflammation in IBD.

The human gut is home to complex microbial ecosystems responsible for one source of human genetic and metabolic diversity. Humans and bacteria have co-evolved; as hosts, we benefit from an adaptive network of organisms for non-nutrient factors, increased digestive capacity, and protection from colonization by pathogens^1^. We coexist synergistically with our gut microbiota, but this relationship can sometimes become pathological^2^. Advancements in sequencing technology now reveal the composition of species that inhabit the human body in both healthy and diseased states. While there exists significant variability in microbiome composition among healthy individuals, the populations of flora found in the healthy human gut appear to be far from random sets of possible microbes, and instead demonstrate significant overlap potentially constituting an essential *core* microbiome^1^.

The specific factors governing the composition of an individual’s microbiome evolve across the lifespan. As a newborn, the microbiome is initially primed through colonization during passage through the birth canal and ingestion of maternal antibodies in breast milk. Throughout life, the microbiome is continually modified by host genetics, diet, and additional exposures such as antibiotic use or parasitic infection. Recent data suggests that most individuals can be clustered into a small number of *enterotypes*, defined as populations with similar microbiome compositions^3^. The particular makeup of one’s microbiome appears to play a critical role in the development of the immune system, especially for T-cell maturation^4^. However, perturbation of the microbiome has been shown to be a major risk factor for opportunistic infections such as *C. difficile* as well as conditions such as obesity and inflammatory bowel disease; in fact, several studies in mice have demonstrated that the microbiome may be a directly causative agent for these diseases^5^,^6^. Although the specific mechanism of pathogenicity is unknown, it has been hypothesized that disruption of the core microbiome may play a pivotal role in the dysregulated immune response present in inflammatory bowel disease (IBD)^7^,^8^.

Previous studies have focused on alterations in abundance levels of microbial species in disease, with less consideration of changes in larger-scale interspecies relationships. As no single organism has been identified as having predominant importance in the pathogenesis of IBD, investigation of the microbial network may be a fruitful approach for identifying the changes in the microbiome responsible for disease. Modeling of species co-occurrence is a common approach used for characterization of microbial ecosystems^9^ across a wide variety of habitats^10^,^11^,^12^,^13^. In recent years this approach has been increasingly applied to the study of the human gut, revealing prominent co-occurrence patterns across individuals^3^,^14^,^15^. Co-occurrence modeling has provided a standard of measurement for assessing the relationship between microbiome composition and host factors such as genetics^16^ and long-term diet^17^. In addition, co-occurrence patterns in the human microbiome have been shown to correlate with predicted metabolic interactions^18^, suggesting that these networks capture relevant ecological relationships among microbes. While co-occurrence modeling has been frequently used for characterization of the microbiome and study of coexistence or competitive exclusion among species^9^, application of advanced network statistics to microbial co-occurrence patterns remains rare. In IBD in particular, network analysis has yet to be widely applied to co-occurrence data despite early suggestions of its utility^19^.

In this work, we seek to characterize and compare the networks of microbiota in patients with IBD and healthy (non-IBD) controls by modeling species co-occurrence (Fig. 1). We characterize these networks using both global metrics and local motif detection, and identify species with significantly altered influence in IBD network structure as estimated by a commonly used centrality measure. To complement these statistical techniques, we also compare healthy and diseased networks using mesoscale analytic techniques, which can offer insights into the structure of clusters of microbes that tend to co-occur with one another relatively consistently over individuals. Specifically, using community detection methods (which are clustering methods developed specifically for networks), we identify clusters of co-occurring species characteristic of the healthy and diseased guts. We hypothesize that these clusters will be distorted in disease, and that alterations will be most prominent in clusters containing a predominance of pathogenic organisms. Such a loss of microbial cluster structure could result in altered gut metabolism, less efficient nutrient processing, and changes in immune education and response^4^. We identify and compare the species serving as inter-cluster hubs in IBD patients and non-IBD controls, with many co-occurrences across clusters, and we hypothesize that species with altered roles within or between the clusters contribute to the development or maintenance of the disease.

Critically, the network organization can have a direct impact on the system’s robustness. Thus, in a final assessment, we study the mutual interconnections of network hubs (a characteristic known as a “rich-club”), and we hypothesize that this structure will be less conducive to interspecies interaction in IBD. Finally, we measure the robustness of each network to simulated attacks in which species are removed to assess their topological role. This simulation technique enables us to compare the predicted fragilities of the healthy and diseased networks and to gain insight into the possible effects of broad-spectrum antibiotic use.

This hierarchical, graph-based analysis of co-occurrence networks in IBD provides a unique perspective for the identification of potential pathogenic species, comparison of microbial communities, and assessment of global network fitness.

## Results

We assess the organization of microbial co-occurrence networks with a battery of statistics drawn from the toolbox of techniques developed in the field of network science. Each assessment addresses a different scale of organization within the networks. Collectively, these assessments offer a comprehensive view of the conserved and variable architectures present in healthy and diseased microbiomes.

### Computation of Global Network Measures

We began by assessing the structure of each network using several global network measures and we compared these measures to those of common benchmark networks. The benchmark networks included (i) a *random* network and (ii) a *lattice* network (see Methods). The values of global network measures for both the real and benchmark networks are found in Table 1.

The clustering coefficient represents the probability that two co-occurring species also co-occur with a third species. We found that both the healthy and IBD networks have average clustering coefficients significantly larger than those of a comparable random network (*p* < 0.001). These results imply that the various microbial populations present in the human gut do not co-occur randomly, but rather that certain bacterial species are highly likely to be found together. This interspecies correlation is present within the IBD population to a lesser extent, suggesting that the composition of multispecies microbial communities characteristic of the healthy gut may be disturbed in the disease state.

That the human gut microbiome is composed of non-random clusters of species is also supported through evaluation of the characteristic path lengths of these networks, defined as the average shortest network distance between two species. Networks in healthy and IBD populations possessed intermediate values of the characteristic path length, with magnitudes greater than those observed in random benchmarks but also less than those observed in lattice networks. A complementary view is offered by the global efficiency, which represents the average inverse shortest path length between two species. As expected, the healthy and IBD networks display global efficiency values that are lower than those observed in the random benchmarks, but higher than those observed in lattice networks. Together, these results support the notion that the human gut microbiome is composed of non-random clusters of species that are highly likely to be found together.

### Motif Detection

To better characterize local patterns of species co-occurrence, we searched each network for motifs composed of 3 species (also known as 3-species motifs) and motifs composed of 4 species (also known as 4-species motifs)^20^. The numbers of motifs of each variety found in each network are shown in Supplemental Fig. 1. In all cases, the motif frequency differed in a statistically significant manner between the real and random networks (*p* < 0.0001 for all differences). These differences are consistent with the finding of significantly higher clustering in the real networks (both IBD and healthy) compared to random; motifs with highly interconnected nodes occur more often in the real networks, while motifs with fewer connections occur more often in the random networks.

However, we observed one motif, marked with a star in Fig. 2, that occurs with significantly higher frequency in the IBD network than in the healthy network. Notably, this motif is solely responsible for the difference in motif profile between healthy and IBD networks (*p* = 0.03 with motif included; no significant difference with motif omitted). This particular motif is interesting in that it contains a fully clustered component (*A* → *B* → *C* → *A*) with an additional, unclustered connection (*C* → *D*), thus representing an intermediate structure uncharacteristic of either the random (generally unclustered) or healthy (generally clustered) networks. The recurrence of this motif in the IBD network demonstrates the local topological derangements responsible for the decreased clustering and modularity of this system, as edges are less exclusively restricted to distinct clusters. Interestingly, the clustering coefficient of this motif (0.583) is similar to the average clustering coefficient of the network. An alternative explanation for this finding is that interpersonal microbial variability is greater among IBD subjects than among healthy subjects. Common occurrence of this motif suggests stratification at the subject level; species *A*, *B*, and *C* cohabitate in some subjects while species *C* and *D* cohabitate in others, with relatively uncommon overlap of these two microbial profiles.

While nearly all organisms participated in this motif more often in IBD than in health, we can identify particular species with the greatest differential participation. Since motif participation is strongly related to node degree, we computed the expected motif count for each species based solely on node degree using a linear regression model. This approach allows us to compute a residual count for each species describing motif participation relative to expectation. Comparing normalized participation counts between the healthy and diseased states reveals that the local connectivity of four species is most strongly skewed toward this motif in IBD. Three of these species are in the *Bifidobacterium genus* (*catenulatum*, *animalis subsp. lactis*, and *bifidum*), while the fourth is *Mitsuokella multiacida*. These species of *Bifidobacteria* are known inflammatory-mediators with probiotic effects in the gut^21^, and may offer therapeutic benefit in IBD^22^. Although the association between *Mitsuokella multiacida* and gut inflammation remains unstudied, this species is increased in the feces of patients with ulcerative colitis^23^. This analysis suggests that the local connectivity of anti-inflammatory species is disproportionately altered in IBD, reflecting the imbalance in pro- and anti-inflammatory drivers characteristic of these diseases.

### Identification of Key Organisms by Eigenvector Centrality

While the previously described results suggest a global topological architecture that is altered in the IBD network, it is possible that these observations could be driven by a few specific organisms, whose pattern of connections have a greater-than-average influence on the network. To test this possibility, we sought to identify specific species with disparate importance in healthy and IBD networks using a measure of species influence referred to as the eigenvector centrality.

We calculated the normalized eigenvector centrality of each species and identified species with the greatest change in centrality between control and diseased networks (Supplemental Fig. 2). The species with the most enhanced centrality in the healthy network are *Akkermansia muciniphilia*, a probiotic organism shown to decrease gut inflammation with potentially protective effects against diabetes and obesity^24^, and *Coprococcus eutactus*, an organism found in greater abundance in healthy controls than in patients with IBD^25^ or IBS^26^. Conversely, the species with most enhanced centrality in IBD are *Catenibacterium mitsuokai*, a microbe implicated in gut inflammation and diet-induced microbiome dysbiosis^27^, and *Bifidobacterium bifidum*, a potent probiotic shown to decrease Th1-driven intestinal inflammation^28^,^29^. The striking map between (a) species identified in this network approach as having altered influence and (b) species described in prior literature as impacting the disease, offers validation of this network-based approach and suggests that the approach might yield additional, not yet studied, microbial targets. Furthermore, the inclusion of *Bifidobacterium bifidum* as more highly influential in IBD illustrates that this network-based analysis provides a perspective that complements pure abundance data; while *Bifidobacterium bifidum* is actually decreased in abundance in IBD^30^,^31^, it becomes more significant in the co-occurrence network, possibly assuming a counter-regulatory role in the inflammatory milieu.

### Computation of Modularity and Consensus Partitions

The results thus far indicate that the global architecture of microbial co-occurence networks differs in diseased individuals, and that key species might play an influential role in observed network function. We now turn to assess these networks from a meso-scale perspective, to better understand the rules governing sets of species that tend to co-occur together with one another.

We begin by studying a mesoscale feature known as *community structure*; networks that display community structure contain sets of nodes that are densely interconnected with one another, more so than expected in an appropriate null model. While many methods exist to identify and characterize community structure, we employ the common modularity maximization approach^32^ (see Methods). Specifically, we calculated the maximum modularity index of the healthy and IBD networks, as well as of benchmark networks (Table 1). Both the healthy and IBD networks possess modularity indices that are significantly greater than those expected in the random network null model (*p* < 0.0001 in each case). This difference supports the hypothesis that the microbiome is characterized by relatively distinct groups of species that tend to co-occur with one another. Notably, the modularity index of the healthy network was significantly greater (*p* < 0.0001) than that of the IBD network, suggesting that the communities of co-occurring organisms present in the healthy gut might be disrupted in the disease state.

It is important to note that the modularity maximization approach is a heuristic approach, and that the optimization of the modularity quality function is traditionally performed many times to ensure an adequate sampling of the modularity landscape^33^. Over 1000 optimizations, we therefore distill a consensus partition in which nodes are assigned to their most likely communities (see Methods). This approach yielded 4 communities in the healthy population and 3 communities in the IBD population (Fig. 3A and Supplemental Fig. 3B). These consensus partitions were fairly representative of the community structure in individual optimizations: in the healthy population, 4 communities were detected in 87% of optimizations, and in the IBD population, 3 communities were detected in 99.9% of optimizations.

The modules of microbial co-occurrence detected in the gut in health and disease may represent clusters of symbiotic organisms, and we can gain insight into the potential function of each module through examination of its species profile. A complete list of the consensus partitions of the organisms in healthy and IBD networks is listed in Supplemental Table 1. In the healthy, non-IBD control network, 4 distinct clusters are identified. Community I (Green, Fig. 3A) is primarily composed of species in the *Bacteroides*, *Bifidobacterium*, *Prevatella*, and *Ruminococcus* genera. These organisms are ubiquitously found in the normal gut microbiome, and make up a significant fraction of the overall microbiome^1^. Community II (Red) consists primarily of *Clostridium* and *Bacteroides* species. These organisms are commonly found in the gut of asymptomatic individuals, although several members of the Clostridium genus are associated with opportunistic gastroenteritis if the microbiome is acutely stressed^34^. Community III (Orange) consists largely of *Enterococcus*, *Streptococcus*, and *Bifidobacteria*, with a smaller section of *Clostridium*. These organisms are primarily commensals, found in the gut of all individuals albeit in lower quantities than the organisms of Community I. Community IV (Blue) consists primarily of *Lactobacillus*, *Proteus*, and *Citrobacter*. These organisms may play an important role in regulating the inflammatory milieu of the gut (see Discussion).

In IBD, three communities are detected. Community I* (Green, Supplemental Fig. 3B) is comparable to Community I from the healthy gut, composed mainly of *Bacteroides*, *Bifidobacterium*, *Prevatella*, and *Ruminococcus*. Community II* (Red) consists primarily of *Lactobacillus* and *Clostridium*, while Community III* (Blue) contains a diverse group of microbes including *Streptococcus*, *Clostridium*, and *Enterococcus*.

### Comparison of Community Structure in Health and Disease

While both co-occurrence networks are characterized by a high degree of modularity, we wished to investigate whether these communities are quantitatively comparable in healthy and diseased populations. To assess similarity between the partitions of species into communities, we use the Rand index^35^, which is a statistical measure that quantifies similarities between partitions of items into sets. We observed that the Rand index between the healthy and IBD consensus partitions was significantly greater than that expected under the null hypothesis of no differences between networks (true Rand index 0.76; Null Rand index obtained by permutation testing was 0.58 ± 0.004; See Supplemental Fig. 4). This calculation indicates that while there are differences in community structure between the healthy and IBD networks, the partitions are still much more similar than would be expected by chance (*p* < 0.0001), implying that much of the global community structure is conserved.

Despite this global similarity, we wished to more accurately quantify the local differences between healthy and IBD partitions. We observed that the perturbation of community structure in disease could be clearly visualized by color-coding nodes (species) of the disease network by community identity in the healthy network (Fig. 3B). Community I is largely preserved in the disease state, implying that the relationship among these microbes is unaffected in IBD. While Community II still shows a high degree of clustering, it is less distinct from Community I in the IBD population than in the healthy population. However, the community structure of Communities III and IV is lost in IBD, as these organisms no longer distinctly co-occur. In healthy individuals, the organisms in these communities do not significantly co-occur with those in the core microbiome; in IBD, however, many of these organisms do co-occur with the core microbes, indicating that they may play a larger role in the IBD gut environment. Community III includes many organisms in the *Enterococcus* and *Bifidobacterium* genera. Several pro-inflammatory species of *Enterococcus* have been shown to induce IBD^40^,^41^, and virulence factors from *Enterococcus* are isolated from patients with IBD^41^. These alterations in bacterial communities are also consistent with direct analysis of patient samples, which show increased populations of *Enterococcus* in patients with IBD^41^. Conversely, *Bifidobacteria* are probiotic in nature and may help to mitigate the pro-inflammatory effects of *Enterococci*^42^. Community IV also consists of organisms with opposing roles in gut inflammation. While *Proteus* and *Citrobacter* bacteria can be associated with inflammation, urinary tract infections, and occasionally gastroenteritis^36^,^37^, *Lactobacillus* is a known probiotic that tends to decrease gut inflammation. In fact, *Lactobacillus* supplementation has been shown to decrease the virulence of both *Proteus*^38^ and *Citrobacter*^39^, and is used both preventatively and therapeutically for individuals with recurrent infections from these organisms. The clustering of these organisms in the healthy network may represent the required balance between pro- and anti-inflammatory factors in healthy, asymptomatic individuals. The loss of clustering in IBD may be in response to the high-inflammation environment, or may play a role in the generation of such an environment.

On closer inspection of this altered community structure, we observed four species that had no significant co-occurrences (degree zero) in the healthy gut, and yet were connected to up to 21% of all other species in IBD. This striking over-connection in IBD suggests a possible role for these organisms in disease. The four species, highlighted in yellow in Fig. 3B, are *Klebsiella pneumonia*, *Campylobacter hominis*, and two species of *Bifidobacter. Klebsiella pneumonia* and *Campylobacter hominis* are potent pro-inflammatory agents: *Klebsiella* has been show to play a major role in the initiation of inflammation in IBD^43^, and *Campylobacter* is a common cause of severe gastroenteritis. The two species of *Bifidobacter* may serve to protect the gut mucosa through inhibition of NF*κ*B^44^, and upregulation of these anti-inflammatory species in IBD is consistent with data from patient fecal samples showing increased populations of probiotics such as *Bifidobacterium* and *Lactobacillus*^45^.

### Network Roles of Microbial Species within Co-Occurrence Communities

In a final assessment of the community structure in healthy and IBD states, we investigated the roles that individual nodes served in the network, as specified by their distributions of intra- and inter-community connections. Specifically, for each node, we calculated two complementary statistics that have previously been used^46^ to define node roles within networks displaying community structure: (i) the intra-module degree *Z*-score, which quantifies a node’s connections within its own community, and (ii) the participation coefficient, which quantifies a node’s preference to connect to nodes in other communities. Nodes that play the role of *connector nodes* display high participation coefficients with relatively low intra-module degree *Z*-scores; conversely, nodes that play the role of *provincial nodes* display high intra-module degree *Z*-scores with relatively low participation coefficients. Note that the inter-modular connections of each species were counted absolutely to prevent spurious findings due to low node degree (Supplemental Fig. 5).

We visualize the role that each species plays in the community structure by mapping the intra-module degree *Z*-score and participation coefficient in a 2-dimensional plane (Fig. 4). In the healthy network, we assign 6 different roles to microbes by tiling the plane according to two rules (i) separating species with *Z* greater than *vs* less than zero, and (ii) separating species with *P* less than 0.2, greater than 0.2 but less than 0.55, and greater than 0.55. These cutoff values mimic those used by Guimerá^46^; the lower cutoff indicates that 90% of a node’s edges are intra-modular, while the upper cutoff indicates that 33% of a node’s edges are inter-modular. We observed that species spanned all 6 roles in both the healthy and IBD networks. However, we also noticed that the roles that species play in the healthy network can be quite different than the roles that the species play in the IBD network (which is evident in Fig. 4 as nodes are color-coded by their role in the healthy network). A particularly interesting finding is shuffling of species with a participation coefficient of zero. We find that connector nodes of the healthy network often lose this role entirely in IBD, reflecting an underlying shift in community structure at a population level. Conversely, many species with no out-of-module co-occurrences in the healthy network demonstrate significant increases in *P* in IBD. These observations indicate that different species are serving as connector nodes in health and disease. Newly important species in the IBD network likely either contribute to the inflammatory environment directly or benefit from elimination of competitive rivals. As such, the unique perspective provided by examination of the network role of individual species may help to identify potentially pathogenic targets for further study.

### Role of Hubs in the Microbial Co-Occurrence Networks

The functional importance of network hubs motivates closer study of their mutual interconnections. We hypothesize that microbial networks are characterized by “rich clubs”, meaning that hub nodes are more likely to be connected to each other than expected based on their degree alone^47^. This structure would suggest that species with high degree comprise a stable core community with common co-occurrence. By investigating the rich club character of the healthy and IBD networks, we can gain insight into the relative structure and robustness of these networks.

Rich-club architecture is often illustrated by curves of the rich-club coefficient (*ϕ*) as a function of the degree *k* (Fig. 5). Yet, the curves from real networks are difficult to interpret without reference to benchmark networks. We therefore also depict in this figure the average rich-club coefficient curves for a set of comparable random networks to model the expected rich-club character based solely on network degree distribution. The ratio of these two curves (real and random) represents the normalized rich-club coefficients for the network under study. This analysis demonstrates that both healthy and IBD networks clearly possess rich club organization, with *ϕ*_*norm*_(*k*) 1, at degrees greater than *k* = 3 (*p* ≤ 0.001). Comparing these two networks, we find that the IBD network possesses a greater rich-club organization at degrees between 15 and 41 (*p* < 0.01 by permutation testing; see Methods), while the healthy network has more rich-club character at degrees above 42 (*p* < 0.01). In this sense, the healthy network possesses a canonical rich-club organization, with a small number of highly interconnected nodes of high degree, while the IBD network contains more of a bourgeoisie-club organization, with a larger number of disproportionately interconnected nodes of slightly lower degree.

### Analysis of Network Robustness

We investigated the effect of the differing network topology in health and disease on network robustness using a technique called *fragility percolation*^48^. In this technique, we iteratively remove nodes from the network and assess changes in the values of network statistics including global efficiency, network diameter, and the size of the largest connected component. The network diameter is defined as the longest of the pairwise shortest path lengths. This method is commonly applied in microbial^49^,^50^,^51^ and ecological^52^,^53^ co-occurrence networks to assess the dependence of global network structure on particular species. While co-occurrence networks do not allow for direct prediction of the impact of network perturbation in an individual, they do provide insight to population-level ramifications of species removal. If a species plays a unique and important role in the network, its removal will significantly impact global network statistics; conversely, the removal of species serving redundant or trivial roles will have little effect. This process was carried out both with random attack - in which nodes are removed with a uniform probability - as well as with targeted attack - in which the node with the highest degree is removed at each step to selectively impact hubs. We use targeted attack to mimic the removal of “keystone species”^54^ that cohabitate with many others and are likely to be particularly important in determining overall network structure.

Overall network structure in both health and disease is strongly robust to random attack, with minimal change in diameter and efficiency until approximately 50% of network nodes have been eliminated and no fragmentation of the largest component until approximately 80% of nodes have been eliminated (Fig. 6A, Supplemental Fig. 6A,B). This finding indicates that, in general, these networks contain sufficient redundancy in interspecies connections to remain stable in structure despite elimination of a significant number of species. Targeted attack on hub regions, however, results in an immediate decline in efficiency in both networks due to the removal of highly connected species (Supplemental Fig. 6C). Interestingly, while both networks experience an increase in diameter with targeted attack, the healthy network exhibits a more rapid rate of increase (Fig. 6B; *p* < 0.001 by permutation testing), with effects seen with as few as 11% of nodes removed (*p* = 0.04). The more rapid increase in diameter of the healthy network under targeted attack reflects the underlying difference in organization of the healthy and diseased states; the non-IBD system is characterized by greater rich-club organization, increasing dependence on hub nodes, resulting in greater fragility under targeted attack. In contrast, the IBD network is less affected due to its less organized initial state, and experiences a change in diameter more similar to that of the random network.

## Discussion

In this study we sought to investigate microbial co-occurrence patterns over a range of network resolutions and to compare these patterns between patients with and without IBD. While previous studies have demonstrated distortion of the microbiome in IBD, the network-based approach presented here offers a unique set of tools for quantifying these changes. We have shown that, on a population level, gut bacteria co-occur in different communities in the disease state. Furthermore, different species assume the roles of network hubs which govern the organization of these communities. Through this work, we provide a novel means of measuring microbiome alterations and establish a comprehensive baseline analysis of such alterations in the present cohort. In addition, we present two methods (eigenvector centrality analysis and participation coefficient measurement) that may prove useful for identification of targets for pathogen-specific studies.

We found that both healthy and IBD gut microbial networks exhibit significant clustering above what would be expected in a random network, indicating that the microbiome is composed of collections of associated species that tend to cohabitate. These organisms may thrive due to synergistic effects on the gut metabolism or immune response, and may generate nutrients or biochemical intermediates on which other species in the cluster depend. It is also possible that these communities form as a result of habitat filtering, and that species tend to co-occur more frequently with other species with which they strongly compete^18^.

We used motif detection to obtain a more detailed view of differences in local connectivity patterns among IBD, healthy control, and random networks. While differences between real and random networks were consistent with expectations based on clustering, we identified one key motif with significantly elevated frequency in IBD networks. This finding reflects disruption of clustering and demonstrates that species, in some cases, differ in local network topology and small-scale clustering in IBD. These species-specific network differences can be further quantified through differential eigenvector centrality. We have shown that this method of analysis is successful in identifying species previously implicated in IBD, suggesting that a graph theoretical perspective may be useful both in corroborating prior literature and generating new suspects for pathogenicity.

The healthy gut microbiome is characterized by well-defined communities of bacteria with strong intra-community co-occurrence relationships, and these communities are variably affected in IBD. One community (Community I) consists of organisms ubiquitously present in the normal gut microbiome. These organisms (*Bacteroides*, *Prevatella*, *Ruminococcus*) compose the bulk of the microbiome, and the relative quantities of these microbes are often used to categorize individuals into certain “enterotypes”, or microbiome profiles^3^,^17^. This community is conserved in IBD, indicating that there may be a “core” microbiome essential for gut function that is resistant to alteration. This observation also suggests that alterations in this community of organisms may not play a major role in the pathogenesis of IBD. However, we have shown that other communities (III and IV, for example), show significant distortion in IBD. These communities are characterized by many bacteria with pro- or anti-inflammatory properties, suggesting that they may mediate and/or respond to the gut inflammatory milieu. In addition, our results indicate that inter-modular connectivity is mediated by different species in IBD. These findings may provide direction for future study into the mechanisms by which certain communities of microbes induce a dysregulated immune response in IBD.

The finding of rich-club organization in the healthy gut microbiome provides insight into the large-scale structure of this system. Just as rich-club regions are suggested to facilitate rapid information transfer in brain networks^55^,^56^, rich-club species may play a crucial role in interspecies communication, allowing for efficient transfer of metabolites and small molecules. These species form a central core of hubs, decreasing the network distance between disparate species. In IBD the exclusivity of the rich-club is reduced, with a much larger number of nodes of lesser degree playing a role. This represents a loss of central organization with increased reliance on more local hubs, supporting our hypothesis that the microbial network in IBD is topologically suboptimal for interspecies interaction. Our finding of rich-club structure in these networks warrants further work to understand the importance of this organization and its distortion in disease.

The rich-club organization of these networks also has implications for network robustness. Our investigation using fragility percolation revealed that both healthy and IBD networks are robust to random attack. However, the healthy network may be more susceptible to targeted attack, with increased diameter and decreased efficiency seen with removal of only a few crucial species. The finding that the global structure of the healthy microbiome network can be perturbed with removal of relatively few key species provides emphasis for the need for judicious use of antibiotics. Antibiotic use, comparable to the simultaneous removal of many nodes, has previously been implicated as a risk factor for development of IBD^57^,^58^. In particular, anti-anaerobic antibiotics, effective against many of the rich-club species removed in the simulated targeted attack on hubs, may contribute to pediatric IBD through disruption of the microbiome^59^,^60^.

### Methodological Considerations

There are several methodological considerations to address. We found that both healthy and IBD gut microbial networks exhibited significant clustering and modularity, above what would be expected in a random network. Some degree of community formation is expected given the method by which the adjacency matrices were defined; among the 154 species in the initial census are many closely related species including 23 species within the genus *Bacteroides*. However, the community structure of this network is not fully explained by these interactions, as each community contains several major genera and species of major genera are also distributed across communities. Thus, the high degree of modularity found in the gut microbiome most likely represents true collections of associated species that tend to cohabitate. Further analysis as to the specific metabolic and immunologic interactions among species within a community is warranted.

It is also important to note several of the limitations of the network models we have generated. While we have demonstrated that the community structure of the microbiome is altered in disease, we cannot distinguish between pathologic and reactionary changes in the network. In addition, our analysis only provides information regarding co-occurrence on a population scale, and does not provide direct information as to the microbial communities present in an individual. Future studies should include comparison of microbial populations among individuals to determine whether subjects from each population cluster based on their microbial profiles. Individuals should also be drawn from a more heterogeneous population than used in this study in order to assess variability within the healthy population as a function of factors such as diet, age, and genetics. Lastly, in this study we used binary edges to indicate co-occurrence above a certain threshold. Future analysis could utilize a weighted network to take into account the strength of co-occurrence between species.

The influence of medication on the microbial network was not assessed due to unavailability of data. All IBD patients were in clinical remission at the time of fecal sampling. Previous research in a mouse model of colitis suggests that while antibiotic use causes significant variation in gut microbial composition, typical IBD therapies including immunomodulation (anti-TNF-*α*) and dietary interventions induce changes of much smaller effect size^61^. This finding has been replicated in human studies showing that non-antibiotic therapies including immunosuppression, NSAIDs (mesalamine), and oral corticosteroids cause only modest effects on overall microbiome alteration, with the most significant impacts being a reduction in *Eschericia*/*Shigella* (associated with mesalamine)^62^,^63^ and a slight increase in *Enterococcus* (associated with mesalamine and immunosuppression)^62^. Although IBD therapeutics may impact microbiome composition, variability in the microbial community network due to treatment is significant less than that associated with interindividual or intercohort variation^64^. As chronic therapy of IBD patients in clinical remission remains unstandardized, it is unlikely that our study captures the effects of any one particular therapy. Further studies should be conducted in order to compare microbial network structure across treatment groups to that of treatment-naive patients.

## Conclusion

In summary, we have characterized the trophic relationships among bacteria in the human gut in individuals with and without IBD through comparison of global and local connectivity and identification of well-defined microbial communities. At the individual species level, we show that local connectivity motifs are altered in IBD and that eigenvector centrality may have utility in implicating novel microbial targets in the pathogenicity of this disease. Global community structure is distorted in IBD, possibly representing an imbalance of pro- and anti-inflammatory organisms, but a core module of organisms is conserved in disease. We have also demonstrated that while the overall degree of inter-modular connectivity is similar in health and disease, the species responsible for inter-modular co-occurrence differ. These alterations in network structure result in a system that has decreased rich-club organization and may be less conducive to metabolic fitness. Finally, we demonstrate that healthy microbiome structure is susceptible to targeted attack on key species. Examination of topological changes in microbial co-occurrence networks provides a unique perspective on the changes of the microbiome in IBD at both global and species-specific scales.

## Methods

### Data Collection and Cohort

This work used data generated by the MetaHIT (Metagenomics of the Human Intestinal Tract) Consortium^65^ and provided by the Borenstein Lab (University of Washington). As part of the multi-institutional MetaHIT project, ninety-nine non-IBD control individuals and twenty-five individuals with inflammatory bowel disease were recruited to characterize the human microbiome^65^. In this study we refer to the non-IBD control population as “healthy”. Total DNA was extracted from fecal samples with an average of 4.5 Gb per sample, providing confidence that most novelty would be captured during sequencing^65^.

### Species Identification

Species populations were identified as part of the MetaHIT project. Illumina Genome Analyzer (GA) technology was used to carry out deep sequencing of total fecal DNA. Illumina GA reads were then aligned with a non-redundant set of 650 sequenced bacterial and archaeal genomes. Using a 90% identity threshold, the proportion of genes covered by the reads that aligned on to only a single position in the set was determined. This approach allows for computation of the normalized species abundance within each individual. The final species population list consists of 154 bacterial species that compose the vast majority of the healthy gut microbiome, and for which whole genome sequence is available and that had sequence coverage 1% in a metagenomic sample from at least 1 of the 124 patients analyzed. This full list is supplied in Supplementary Table 1. More details regarding the methods of data collection and analysis can be found in Qin, *et al.*^65^.

### Construction of Co-occurrence Adjacency Matrix Space

**Figure 1:**
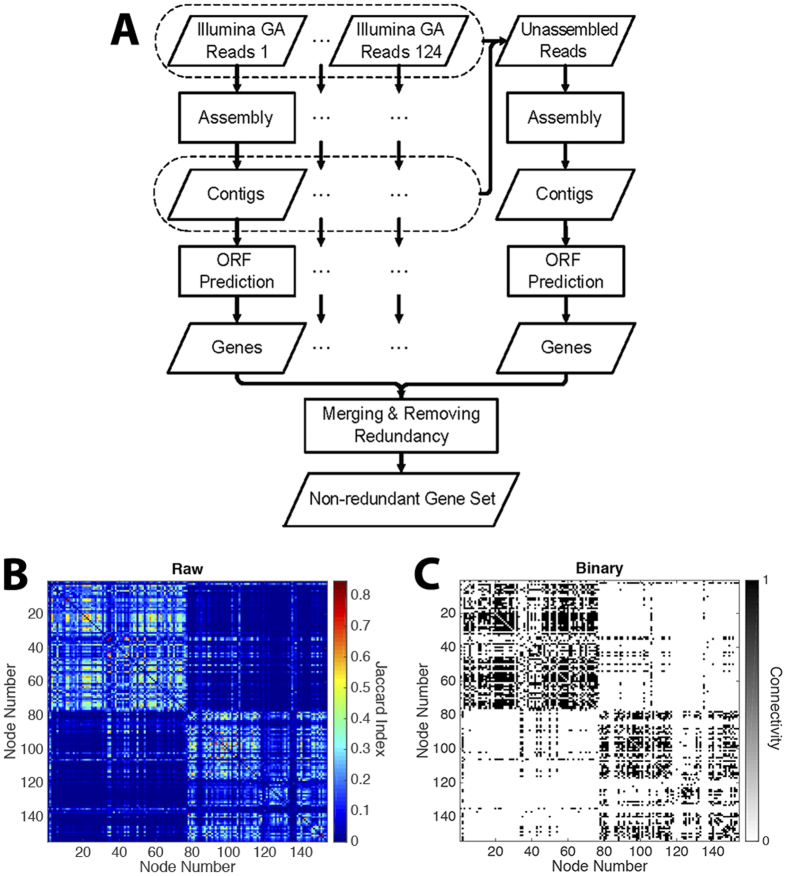
Microbial co-occurrence networks. (**A**) Fecal samples harvested from healthy subjects and subjects with IBD. Organisms identified by total DNA deep sequencing and alignment with reference genomes. (**B**) Co-occurrence matrices generated from abundance data using Jaccard similarity. The raw co-occurrence matrix containing weighted data from healthy population is shown. (**C**) Healthy co-occurrence matrix after thresholding to isolate the strongest 20% of connections. For additional details regarding data and computational methods, see Methods. Panel (**A**) reprinted by permission from Macmillan Publishers Ltd: *Nature*, Qin, *et al.*^65^, copyright 2010.

**Figure 2:**
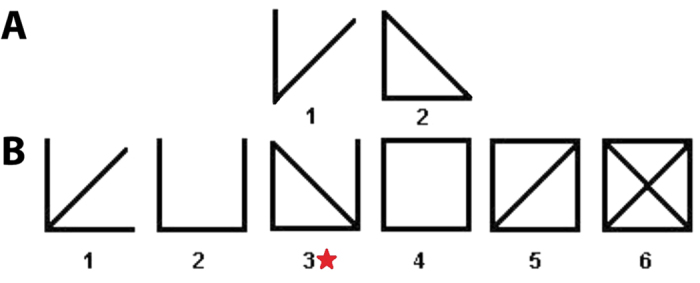
Motifs in microbial co-occurrence networks. Illustration of bidirectional motifs of 3 (**A**) and 4 (**B**) nodes. IBD networks have a significantly higher frequency of 4-node motif number 3.

**Figure 3:**
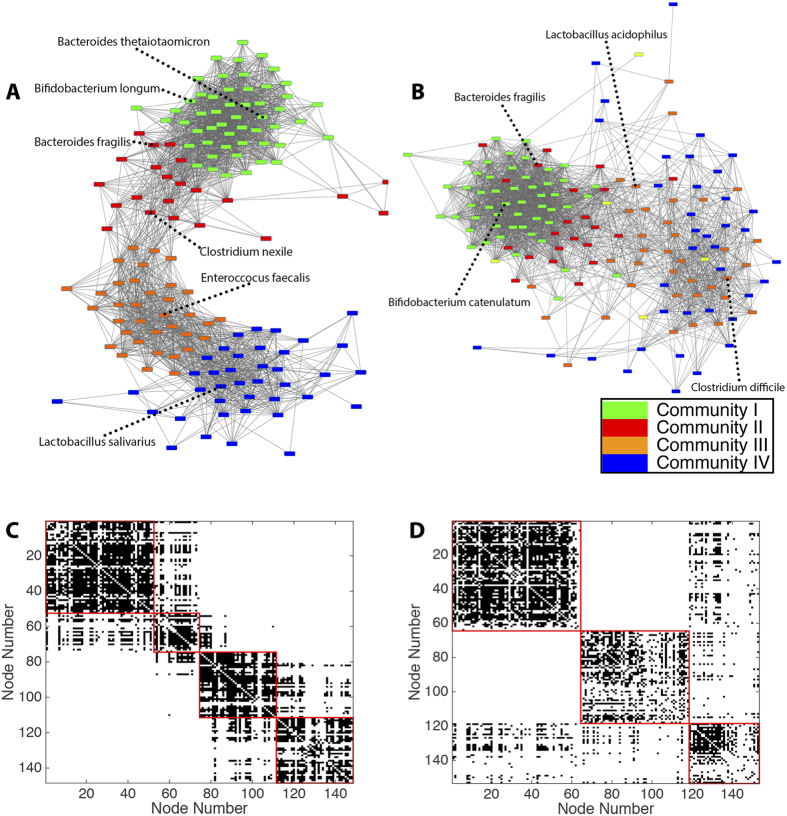
Identification of Microbial Communities in Health and Disease. (**A**) Healthy network color-coded by consensus partition. Community I = Green; Community II = Red; Community III = Orange; Community IV = Blue. Representative, high-degree species from each module are labeled. (**B**) IBD network color-coded by consensus partition of healthy network. (**C**) Heat map of control network and (**D**) Heat map of IBD network with nodes arranged according to consensus partition.

**Figure 4:**
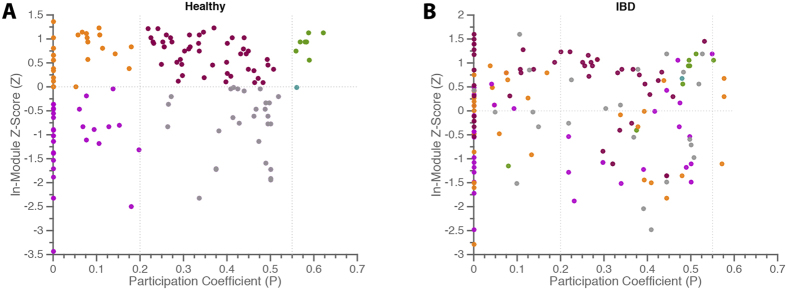
Network Roles of Microbial Species within Co-Occurrence Communities. Stratification of node roles in healthy (**A**) and IBD (**B**) networks using the intra-module degree *Z*-score, *Z*, and the participation coefficient, *P*. Nodes are color coded by their role in the healthy network.

**Figure 5:**
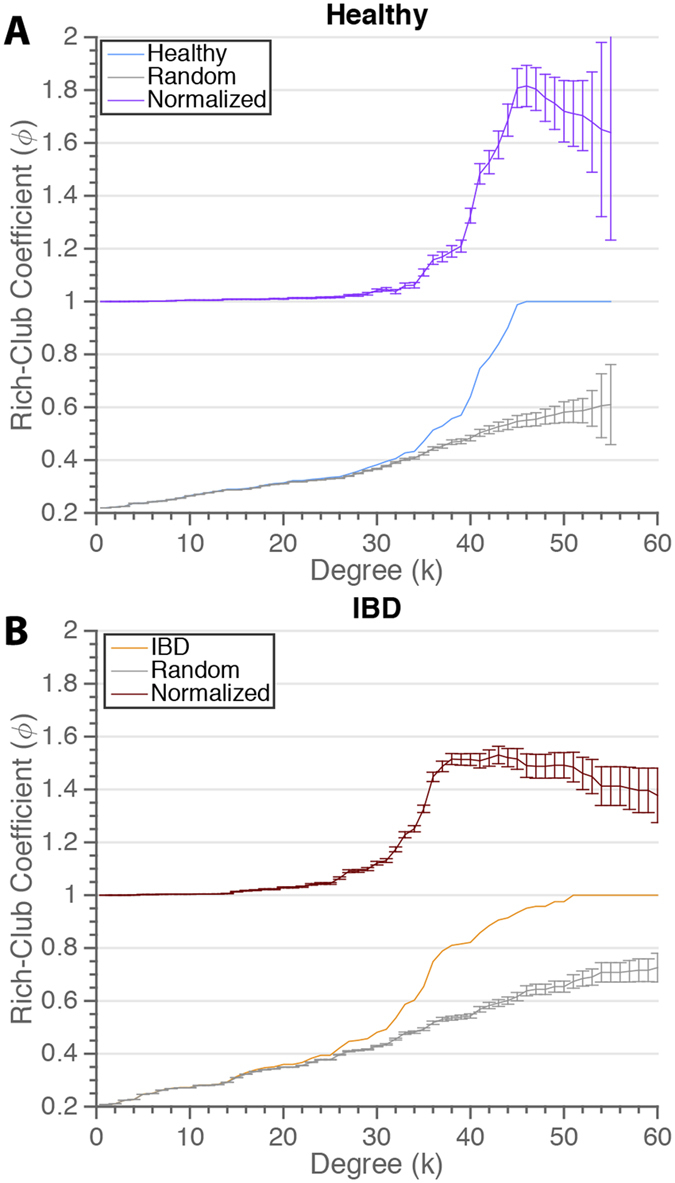
Role of Hubs in the Microbial Co-Occurrence Networks. Rich-club coefficients in healthy (**A**) and IBD (**B**) networks as a function of minimum node degree. The normalized coefficient curve is the ratio of coefficient curves of the real networks and comparable random networks. A normalized coefficient greater than one indicates more rich-club organization that expected by chance.

**Figure 6:**
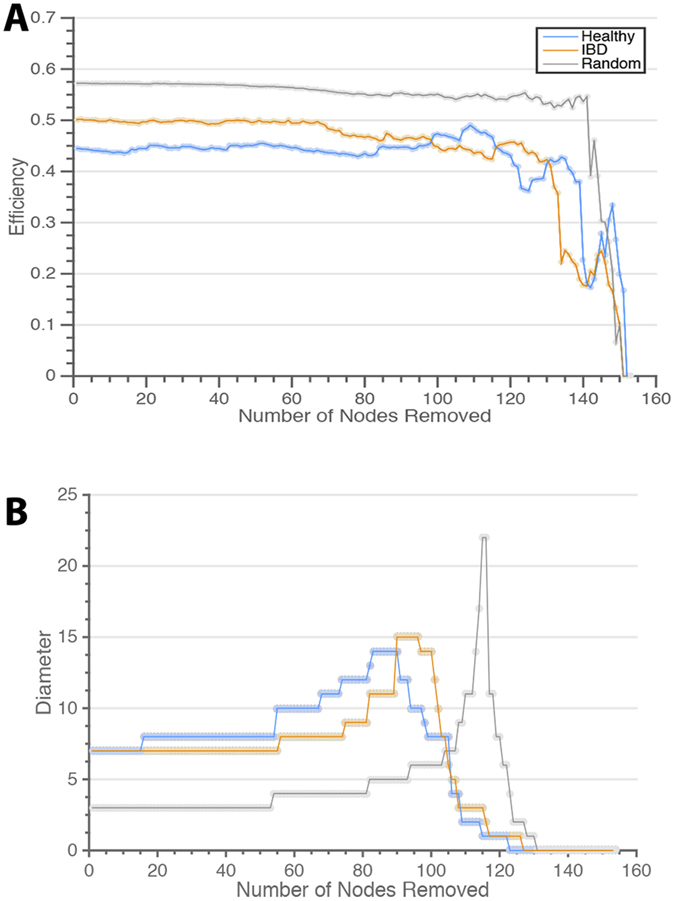
Network Robustness and Resilience. (**A**) Global network efficiencies measured as nodes are randomly removed from the networks. Network efficiencies are robust to random attack. (**B**) Network diameter measured as the highest-degree nodes are removed. Network diameter is affected by targeted attack, especially in the healthy network.

A co-occurrence adjacency matrix was constructed by calculating the Jaccard similarity index between pairwise species^18^,^66^. This method was developed to maintain the influence of relative species abundance in the calculated index. The resulting value exists between 0 (no co-occurrence in any samples) and 1 (perfectly overlapping profiles of species occurrence). These similarity indices were compiled into an adjacency matrix as shown in the heat map in Fig. 1. While this matrix provides weighted values indicating the degree of co-occurrence between two species, our analysis was performed on a binary, undirected, thresholded version of this matrix. The threshold was chosen to preserve connections with Jaccard similarity greater than one standard deviation above the mean in order to ensure that these connections are biologically significant and to limit the impact of statistical noise. As a result, the final matrix used for analysis contained approximately the strongest 20% of co-occurrences. Robustness of our results to the chosen threshold is demonstrated in Supplemental Fig. 7. In the case of the healthy population, 6 species became individually disconnected; similarly, in the IBD population 1 species became disconnected. Thus, the resulting healthy and IBD networks contained 148 and 153 connected nodes, respectively, representing each bacterial species, connected by 2356 edges, representing significant co-occurrence after thresholding. The degree of each node is defined as the number of edges connected to that node. Species were included in the analysis if they maintained at least one significant connection in at least one network. Network analyses was carried out using a combination of the Brain Connectivity Toolbox^67^ and in-house software.

### Generation of Benchmark Null Models

Null models were generated for the control and disease networks through randomization of edges with preserved degree distribution. It is important to consider that our experimental networks are constructed using Jaccard similarity. The Jaccard similarity between nodes *i* and *k*, *J*_*s*_(*i*, *k*), can be related to a distance metric, 1 − *J*_*s*_(*i*, *k*), such that triplets of connected nodes must satisfy the triangle inequality. While we did not impose this constraint during null model design, review of 1000 null networks demonstrated that the triangle inequality was violated in only 0.4 ± 0.08% of triplets, suggesting that the omission of this criterion had a negligible effect on global network characteristics such as the clustering coefficient or rich club coefficient.

### Measuring Influence with Eigenvector Centrality

Eigenvector centrality is a characteristic of network nodes that provides a more sophisticated view of local connections than the node degree by also considering the degree of a node’s neighbors. This approach accounts for the fact that all edges are not equal: connections to a highly connected node may render a node more significant or influential. We define the centrality of node *i*, *x*_*i*_, as a proportion of the average centrality of its neighbors as


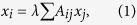


where *λ* is a constant. Defining the vector of centralities *x* = (*x*_1_, *x*_2_, ...), we can rewrite this equation in matrix form as


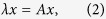


thereby demonstrating that *x* is an eigenvector of the adjacency matrix with eigenvalue *λ*. To ensure that centrality values are non-negative, *λ* must be the largest eigenvalue of *A* and *x* must be the corresponding eigenvector^68^,^69^.

### Identifying Microbial Clusters: Modularity Optimization

We investigated whether the microbial co-occurrence networks displayed modular characteristics by determining their community structure via modularity maximization using a Louvain-like locally greedy algorithm^32^,^70^. This method creates subdivisions into non-overlapping groups of nodes in a locally greedy manner that maximizes within group edges in comparison to an appropriate null model. Specifically, nodes are allocated to modules (also known as *communities*) by a greedy algorithm that maximizes the modularity index *Q*, defined as:


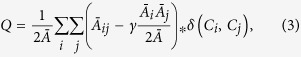


where *A*_*ij*_ is the adjacency matrix, *A*_*i*_ is the strength of node *i*, 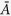 is the average strength of the network, *C*_*i*_ is the community to which node *i* is assigned and *γ* is a scaling parameter used to adjust the size of the modules (see supplemental section on Determination of Community Resolution). To account for the near degeneracy of the modularity landscape^33^, the optimization was repeated 1000 times, and we calculated the mean maximum modularity of the network. In the case of the random network benchmark, modularity maximization was applied 100 times to each of 100 generated networks.

### Regional Intra- and Inter-Module Connectivity

We sought to investigate the potential role of each individual species by examining its intra-module connectivity and its inter-module connectivity^46^. We hypothesized that species with many connections across modules may be important in regulating global network dynamics. We determined the intra-module degree *Z*-score and participation coefficient of each node. The intra-module degree *Z*-score, *Z*_*i*_, measures the degree of intra-module connectivity of node *i* relative to other nodes in the module and is defined as:


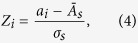


where *a*_*i*_ is the number of intra-module connections of node *i*, 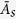 is the mean number of intra-modular connections in module *s*, and *σ*_*s*_ is the standard deviation. A high value of *Z*_*i*_ indicates strong intra-modular connectivity of node *i*. Inter-modular connectivity was measured using the participation coefficient, defined as:


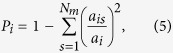


where *a*_*is*_ is the number of connections of node *i* in module *s* and *a*_*i*_ is the total number of connections of node *i*. The participation coefficient will be close to zero if almost all connections are intra-modular, and will be closer to one if a greater proportion of connections are inter-modular.

### Rich-Club Organization

The rich-club coefficient was calculated as a function of node degree (*k*) using the Brain Connectivity Toolbox^67^. For each value *k*, all nodes with degree less than or equal to *k* were removed from the network. For the remaining network, the rich-club coefficient *ϕ*(*k*) is calculated as the ratio of the number of existing edges to the total number of possible edges in the reduced network^47^. Formally, the rich-club coefficient is given by


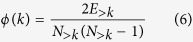


where *E*_>*k*_ is the number of edges in the reduced network and *N*_*k*_ is the maximum possible number of edges.

The rich-club coefficient curve for the random benchmark networks was generated by averaging results from 1000 random networks with preserved degree distribution. To determine the statistical significance of rich-club organization, permutation testing was used^71^. In constructing comparable random networks, we generated a null distribution of rich-club coefficients from random topologies. The one-sided *p*-value was calculated over a range of *k* by measuring the percentage of *ϕ*_*random*_ that exceeded *ϕ*. Statistical comparison of the normalized rich-club curves in control and disease networks was carried out using functional data analysis^72^. For each *k*, the difference between the curves was calculated. Individual observations from each group were then randomly reassigned in order to determine the expected mean and standard deviation of this difference under the null hypothesis. This distribution was used to generate a one-sided *p*-value for the observed difference between the curves.

## Additional Information

**How to cite this article**: Baldassano, S. N. and Bassett, D. S. Topological distortion and reorganized modular structure of gut microbial co-occurrence networks in inflammatory bowel disease. *Sci. Rep.* **6**, 26087; doi: 10.1038/srep26087 (2016).

## Supplementary Material

Supplementary Information

## Figures and Tables

**Table 1:** Global network measures.

Network	*C*	*PL*	*E*	*Q*
Healthy	0.679	2.86	0.482	0.480 ± 0.003
IBD	0.596	2.47	0.508	0.411 ± 0.005
Random	0.218	1.77	0.608	0.123 ± 0.004
Lattice	0.762	6.45	0.288	0.663 ± 0.003

*C* = average clustering coefficient; *PL* = characteristic path length; *E* = global efficiency; *Q* = modularity index.
