# Supplementary material for: Topological distortion and reorganized modular structure of gut microbial co-occurrence networks in inflammatory bowel disease
Source: Sci Rep. 2016 May 18;6:26087. doi: 10.1038/srep26087 (PMC4870640; doi:10.1038/srep26087)

Topological distortion and reorganization of modular structure of gut microbial co-occurrence networks in inflammatory bowel disease

Steven Baldassano[1,3] and Danielle S. Bassett[1,2,3]

[1]Department of Bioengineering, University of Pennsylvania, Philadelphia, PA 19104 USA

[2]Department of Electrical & Systems Engineering, University of Pennsylvania, Philadelphia, PA 19104 USA

[3]Center for Neuroengineering and Therapeutics, University of Pennsylvania, Philadelphia, PA 19104

Supplemental Materials

Supplemental Table 1:

List of species names and reference genomes with their community assignments in the healthy (non-IBD control) and IBD networks. Note that numerical values of the community assignments are arbitrary and not equivalent between networks. They should be treated as categorical and not ordinal variables. Species with no significant co-occurrences in a network are listed as N/A.

	Species Name/Reference Genome	Healthy Community	IBD Community
1	Escherichia_coli_O157_H7_EC4115	2	1
2	Leuconostoc_mesenteroides_mesenteroides_ATCC_8293	4	3
3	Coprococcus_comes_ATCC_27758	1	1
4	Akkermansia_muciniphila_ATCC_BAA_835	1	1
5	Alistipes_putredinis_DSM_17216	1	1
6	Anaerotruncus_colihominis_DSM_17241	2	2
7	Bacteroides_caccae_ATCC_43185	1	1
8	Bacteroides_cellulosilyticus_DSM_14838	1	1
9	Bacteroides_coprocola_M16_DSM_17136	1	1
10	Bacteroides_coprophilus_DSM_18228	2	2
11	Bacteroides_dorei_5_1_36_D4	1	1
12	Bacteroides_dorei_DSM_17855	2	1
13	Bacteroides_eggerthii_DSM_20697	1	1
14	Bacteroides_finegoldii_DSM_17565	1	1
15	Bacteroides_fragilis_3_1_12	2	1
16	Bacteroides_intestinalis_341_DSM_17393	1	2
17	Bacteroides_ovatus_ATCC_8483	1	1
18	Bacteroides_pectinophilus_ATCC_43243	1	1
19	Bacteroides_plebeius_M12_DSM_17135	1	1
20	Bacteroides_sp_2_1_7	1	1
21	Bacteroides_sp_2_2_4	1	1
22	Bacteroides_sp_4_3_47FAA	1	1
23	Bacteroides_sp_9_1_42FAA	1	1
24	Bacteroides_sp_D1	1	2
25	Bacteroides_stercoris_ATCC_43183	1	1
26	Bacteroides_thetaiotaomicron_VPI_5482	1	1
27	Bacteroides_uniformis_ATCC_8492	1	1
28	Bacteroides_vulgatus_ATCC_8482	1	1
29	Bacteroides_xylanisolvens_XB1A	1	1
30	Bifidobacterium_adolescentis_L2_32	1	1
31	Bifidobacterium_catenulatum_DSM_16992	2	1
32	Bifidobacterium_longum_longum_CCUG_52486	1	1
33	Bifidobacterium_pseudocatenulatum_DSM_20438	1	2
34	Blautia_hansenii_VPI_C7_24_DSM_20583	3	1
35	Bryantella_formatexigens_I_52_DSM_14469	3	3

36	Butyrivibrio_crossotus_DSM_2876	1	2
37	Catenibacterium_mitsuokai_DSM_15897	2	1
38	Clostridiales_sp_SS3_4	1	1
39	Clostridium_asparagiforme_DSM_15981	2	2
40	Clostridium_bartlettii_DSM_16795	2	2
41	Clostridium_bolteae_ATCC_BAA_613	2	1
42	Clostridium_leptum_DSM_753	2	1
43	Clostridium_methylpentosum_R2_DSM_5476	3	2
44	Clostridium_nexile_DSM_1787	2	2
45	Clostridium_scindens_ATCC_35704	3	2
46	Clostridium_sp_L2_50	1	1
47	Clostridium_sp_M62_1	2	1
48	Clostridium_sp_SS2_1	1	1
49	Collinsella_aerofaciens_ATCC_25986	1	1
50	Coprobacillus_sp_D7	3	2
51	Coprococcus_eutactus_ATCC_27759	1	1
52	Dorea_formicigenerans_ATCC_27755	1	1
53	Dorea_longicatena_DSM_13814	1	1
54	Enterococcus_faecalis_TX0104	3	2
55	Eubacterium_biforme_DSM_3989	1	1
56	Eubacterium_hallii_DSM_3353	1	1
57	Eubacterium_rectale_M104_1	1	1
58	Eubacterium_siraeum_70_3	1	1
59	Eubacterium_ventriosum_ATCC_27560	1	1
60	Faecalibacterium_prausnitzii_SL3_3	1	1
61	Gordonibacter_pamelaeae_7_10_1_bT_DSM_19378	2	2
62	Holdemania_filiformis_VPI_J1_31B_1_DSM_12042	2	2
63	Parabacteroides_distasonis_ATCC_8503	1	1
64	Parabacteroides_johnsonii_DSM_18315	1	1
65	Parabacteroides_merdae_ATCC_43184	1	1
66	Prevotella_copri_CB7_DSM_18205	1	1
67	Pseudoflavonifractor_capillosus_ATCC_29799	2	2
68	Roseburia_intestinalis_M50_1	1	1
69	Ruminococcus_bromii_L2_63	1	1
70	Ruminococcus_gnavus_ATCC_29149	2	1
71	Ruminococcus_lactaris_ATCC_29176	1	1
72	Ruminococcus_obeum_A2_162	1	1
73	Ruminococcus_sp_SR1_5	1	1
74	Ruminococcus_torques_L2_14	1	1
75	Streptococcus_thermophilus_LMD_9	1	1
76	Subdoligranulum_variabile_DSM_15176	2	2
77	Clostridium_symbiosum_WAL_14163	2	1
78	Actinobacillus_pleuropneumoniae_sv_7_AP76	4	3

79	Actinomyces_odontolyticus_ATCC_17982	3	3
80	Anaerococcus_hydrogenalis_DSM_7454	4	3
81	Anaerofustis_stercorihominis_DSM_17244	3	3
82	Anaerostipes_caccae_DSM_14662	3	2
83	Bifidobacterium_angulatum_DSM_20098	3	3
84	Bifidobacterium_animalis_lactis_AD011	N/A	1
85	Bifidobacterium_bifidum_NCIMB_41171	N/A	1
86	Bifidobacterium_breve_DSM_20213	3	2
87	Bifidobacterium_dentium_ATCC_27678	3	2
88	Bifidobacterium_gallicum_DSM_20093	4	3
89	Blautia_hydrogenotrophica_DSM_10507	3	2
90	Butyrivibrio_fibrisolvens_16_4	3	3
91	Campylobacter_concisus_13826	4	3
92	Campylobacter_hominis_ATCC_BAA_381	N/A	3
93	Candidatus_Sulcia_muelleri_GWSS	3	3
94	Citrobacter_koseri_ATCC_BAA_895	4	3
95	Citrobacter_sp_30_2	4	3
96	Clostridium_difficile_630	3	3
97	Clostridium_hylemonae_DSM_15053	3	3
98	Clostridium_perfringens_ATCC_13124	4	3
99	Clostridium_phytofermentans_ISDg	3	3
100	Clostridium_ramosum_VPI_0427_DSM_1402	3	2
101	Clostridium_sp_7_2_43FAA	3	3
102	Clostridium_spiroforme_DSM_1552	2	2
103	Collinsella_intestinalis_DSM_13280	3	3
104	Collinsella_stercoris_DSM_13279	3	2
105	Cronobacter_sakazakii_ATCC_BAA_894	4	3
106	Desulfovibrio_piger_ATCC_29098	1	1
107	Desulfovibrio_vulgaris_Miyazaki_F	3	3
108	Enterobacter_cancerogenus_ATCC_35316	4	3
109	Enterobacter_sp_638	4	3
110	Enterococcus_faecalis_TX1322	3	3
111	Enterococcus_sp_7L76	3	3
112	Escherichia_fergusonii_UMN026_ATCC_35469	3	3
113	Eubacterium_dolichum_DSM_3991	3	2
114	Finegoldia_magna_ATCC_29328	4	3
115	Fusobacterium_nucleatum_nucleatum_ATCC_25586	4	2
116	Haemophilus_influenzae_NTHi_86_028NP	3	3
117	Haemophilus_parasuis_SH0165	4	3
118	Helicobacter_pullorum_MIT_98_5489	4	2
119	Klebsiella_pneumoniae_342	N/A	3
120	Lactobacillus_acidophilus_NCFM	3	2
121	Lactobacillus_casei_casei_BL23	4	2

122	Lactobacillus_delbrueckii_bulgaricus_ATCC_11842	3	2
123	Lactobacillus_fermentum_IFO_3956	4	3
124	Lactobacillus_gasseri_ATCC_33323	4	3
125	Lactobacillus_helveticus_DPC_4571	4	3
126	Lactobacillus_hilgardii_ATCC_8290	4	3
127	Lactobacillus_johnsonii_NCC_533	4	3
128	Lactobacillus_paracasei_ATCC_25302	4	3
129	Lactobacillus_reuteri_SD2112_ATCC_55730	4	3
130	Lactobacillus_sakei_sakei_23K	4	2
131	Lactobacillus_salivarius_HO66_ATCC_11741	4	2
132	Lactobacillus_ultunensis_DSM_16047	4	3
133	Lactococcus_lactis_cremoris_MG1363	3	3
134	Megamonas_hypermegale_ART12_1	N/A	1
135	Methanobrevibacter_smithii_DSM_2375	2	2
136	Methanosphaera_stadtmanae_DSM_3091	N/A	N/A
137	Mitsuokella_multiacida_DSM_20544	2	2
138	Pasteurella_multocida_multocida_Pm70	4	3
139	Pediococcus_pentosaceus_ATCC_25745	4	3
140	Porphyromonas_gingivalis_ATCC_33277	3	3
141	Proteus_mirabilis_HI4320	4	3
142	Proteus_penneri_ATCC_35198	4	3
143	Pseudomonas_aeruginosa_LESB58	4	3
144	Salmonella_enterica_sv_Heidelberg_SL476_CVM30485	4	3
145	Staphylococcus_saprophyticus_saprophyticus_ATCC_15305	4	3
146	Streptococcus_gordonii_Challis_CH1	3	2
147	Streptococcus_infantarius_infantarius_ATCC_BAA_102	3	2
148	Streptococcus_mutans_UA159	3	3
149	Streptococcus_pneumoniae_Hungary19A_6	3	2
150	Streptococcus_pyogenes_M4_MGAS10750	4	3
151	Streptococcus_sanguinis_SK36	3	3
152	Streptococcus_suis_05ZYH33	4	3
153	Thermoanaerobacter_sp_X514	4	3
154	Tropheryma_whipplei_Twist	4	3

Supplemental Text:

Methods:

Network Analysis and Reference Networks

Network characteristics including clustering coefficient, path length, efficiency, modularity, and degree distribution were computed using the Brain Connectivity Toolbox (BCT)[67]. The BCT was also used for the generation of benchmark null model networks for statistical comparison. We chose random and lattice networks as benchmark null models to represent two extremes of network clustering; a random network has very little clustering and a lattice network has very high clustering. The random and lattice networks were designed to match the number of nodes and the density of edges observed in the healthy control network. We generated 100 random networks and we report the averaged values of network statistics over this ensemble. In the case of the lattice, the constraint on edge number prevented generation of a perfectly regular lattice; once a regular lattice was generated, additional edges were randomly interspersed to connect nodes that share a neighbor in common (i.e. the additional connection in a network 1→2→3 would connect 1→3).

Motif Detection

Three- and four-node motifs were detected using the BCT [67]. Motifs were determined to occur with statistically significant frequency by comparison to baseline values obtained from averaging motif frequencies from 1,000 randomly rewired networks with preserved node degree. Motif frequency in control networks was not significantly affected by preservation of healthy *vs.* IBD network degree distribution. All edges were considered to be bidirectional, resulting in 2 unique 3-node motifs and 6 unique 4-node motifs.

Determination of Community Resolution

It is important to consider that the resolution of communities detected by the Louvain-like locally greedy algorithm utilized here can be controlled by altering the value of γ, known as the structural resolution parameter [73]. Larger values of γ result in smaller (higher resolution) communities; smaller values of γ result in larger (lower resolution) communities. In this data, the maximum modularity index of the network is inversely related to the resolution at which communities are determined (Supplemental Figure 8a), as larger, lower-resolution communities allow for an increased proportion of within-community edges.

A traditional choice for the value of the structural resolution parameter is unity [73]. We therefore chose to use this value, but we also validated this choice by examination of the specific structure in our data. Specifically, we determined the community structure at a range of γ values between 0.1 and 3.0 in increment of 0.5, and we visualized the resulting communities on an adjacency heat map (Supplemental Figure 8b-d). Using this information, we chose a γ value of 1 for subsequent analysis, as this value captured the modular appearance of the network while still maintaining the vast majority of edges within communities. The choice of one as the value of γ was also justified by the resultant definition of communities in the healthy group, consistent with the

community structure obtained by deletion of high edge betweenness centrality connections [74](See below).

Community Detection By Edge Betweenness Centrality

We wished to validate the communities determined by the Louvain method by identifying community structure through a different approach for comparison. The second algorithm we chose is a popular method that identifies communities based on a network statistic known as the edge betweenness centrality [74]. This approach relies on the concept that inter-community edges are likely to be characterized by high betweenness centrality values, as these paths must be traversed to connect nodes in differing modules. In this method, communities were identified through repetitive identification and deletion of the highest edge betweenness centrality connections [74]. The betweenness centrality of each edge was determined using the BCT, and the connection with the highest betweenness centrality was removed. Importantly, the edge betweenness centrality values were recalculated after each reduction. This technique was repeated until all "outlier" edges with betweenness centrality greater than 2 standard deviations from the mean were removed. At this point, each cluster of nodes with persistent connections is defined as a unique community. The community structure of the healthy network as obtained through betweenness-centrality-based detection is shown in Supplemental Figure 3A. This approach yields almost exactly the same community structure as optimization of modularity. The communities determined by this method differ only in that the smallest community recognized by our consensus detection is not determined to be a separate module, while the exact structure of the remaining two modules is maintained. Therefore, the specific community structure of these networks is robust to the method of community detection.

Comparison of Community Structure

Community structure was compared between networks by determining the Rand similarity index [35]. This index is a measure of how likely two nodes are to be categorized in the same manner (either in the same network or in different networks) across conditions. A Rand value of 0 indicates that the two networks do not agree on any pair of nodes, while a Rand value of 1 indicates that the networks are clustered identically. To determine the statistical significance of a given Rand value, a null model was generated for each comparison using permutation testing. When comparing structure between two groups with different numbers of communities, permutation testing was carried out such that the community assignments of one partition were randomly rearranged while the number of communities was conserved. This approach generates a Rand index for the null model that takes into account the inherent difference in community structure due to different numbers of communities alone.

Results:

Evaluation of Network Degree Distribution

The networks were also characterized through measurement of the degree of each node, summarized in the degree distribution histograms in Supplemental Figure 9. The IBD and healthy networks both approximate the random degree distribution, but are skewed to have a large number of very low degree nodes (degree < 10) as well as several high degree nodes (degree approximately 30). This distribution supports the notion that many species occur in trophically local clusters, commonly occurring with only a few other species (low degree), while some species serve as hubs, allowing for co-occurrence of several small niches. In this way, the gut microbiome degree distributions, in both healthy and disease states, are better modeled as small world networks with a wider variation in degree than as random networks with its narrower degree distribution. However, the microbiome distribution in IBD lacks a clear peak at the expected average degree of 16 and features a preponderance of low-degree nodes, possibly indicating a state of increased community disorder with many sparsely connected species.

As expected, the degree distribution for the lattice network approximates the delta function at the average degree of 16. The distribution varies slightly from a pure delta function due to the constraint on edge density, preventing construction of a perfectly regular lattice. The random network degree distribution is approximated by a binomial distribution centered at the average degree.

References

[35] Traud, A, Kelsic, E, Mucha, P, Porter, M. Comparing Community Structure to Characteristic Social Networks. SIAM Rev. 53(3), 526-543 (2011).

[67] Rubinov M, Sporns O. Complex network measures of brain connectivity: Uses and interpretations. NeuroImage 52, 1059-69 (2010).

[73] Bassett, Danielle, et al. Robust detection of dynamic community structure in networks. Chaos. 23(1): 013142 (2013).

[74] Girvan, M., and Newman, M.E.J. Community structure in social and biological networks. PNAS. 99(12) 7821-7826 (2002).

Supplemental Figure 1:

Note that motif codes correspond to diagrams presented in Figure 2 of the text. The key motif with significantly different frequency in IBD is highlighted with a red box in panel B.

A. Frequency of 3-node motifs

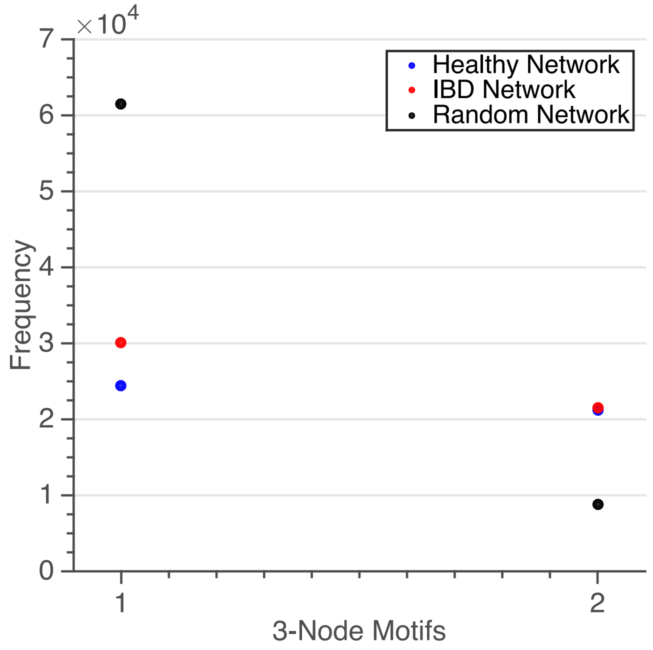

B. Frequency of 4-node motifs

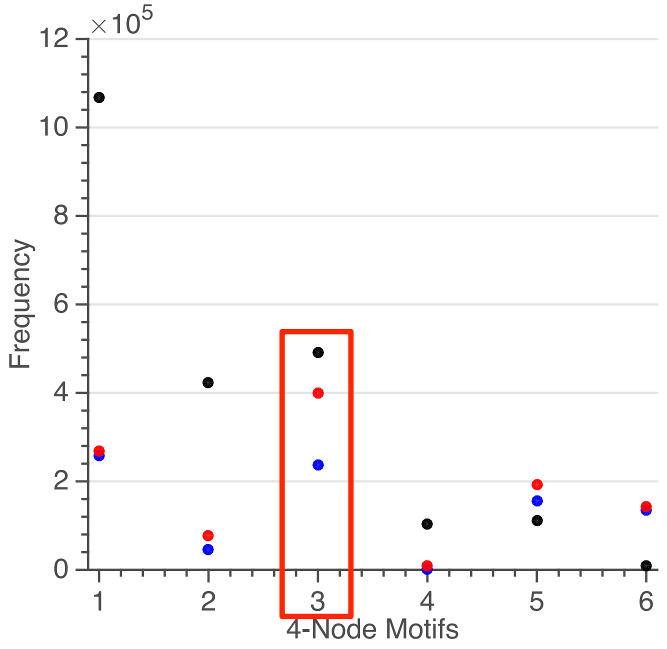

Supplemental Figure 2:

Difference in normalized eigenvector centrality for each node between healthy and IBD networks. Positive values indicate higher eigenvector centrality in the healthy network. Highlighted nodes correspond to outlier species discussed in the text.

A.

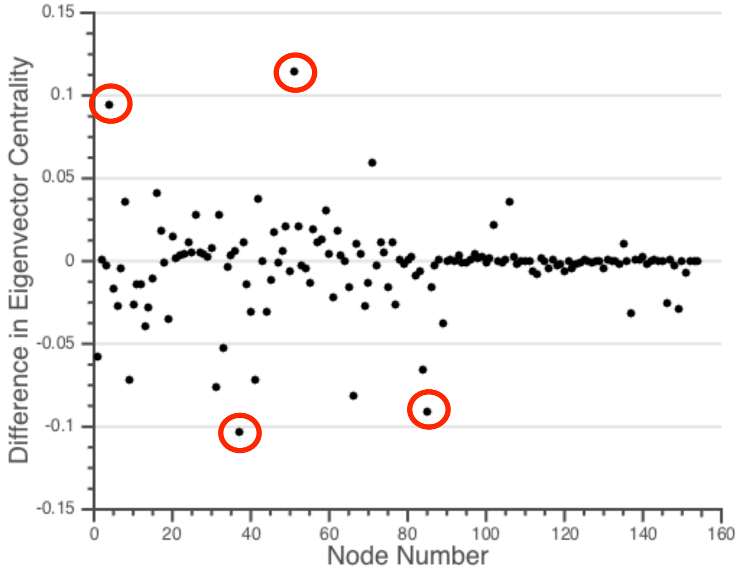

For comparison, the absolute eigenvalue magnitudes are shown for the control (blue) and disease (red) networks. Data associated with the noted differences above are highlighted with arrows.

B.

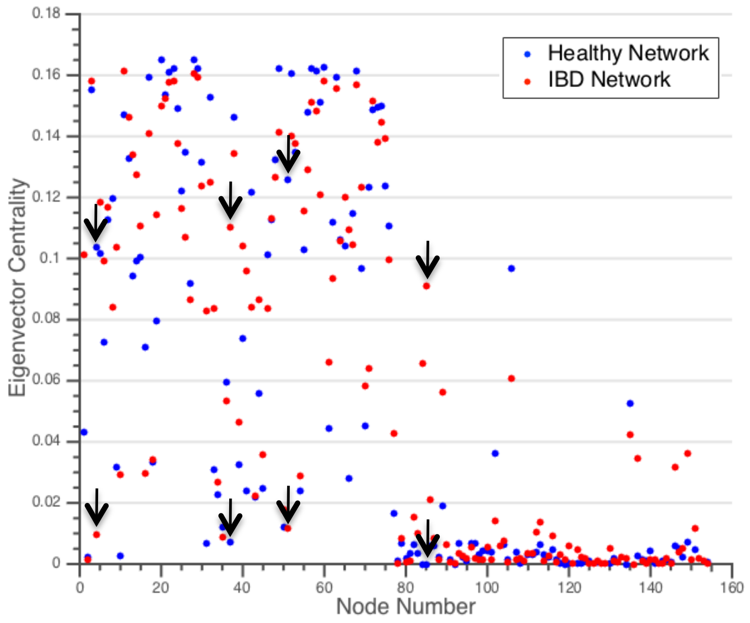

Supplemental Figure 3:

A. Healthy network where each node is color-coded according to the community to which it is assigned; the partition of nodes into communities was determined by removal of edges with high betweenness centrality.

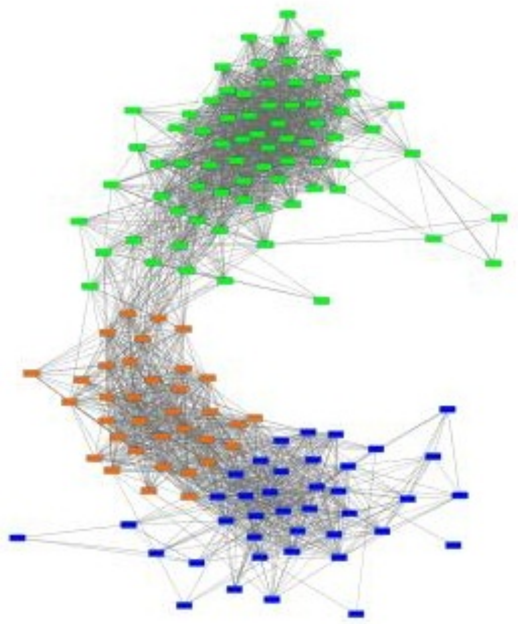

B. IBD network where each node is color-coded according to the community to which it is assigned; the partition of nodes into communities was determined by the Louvain-like locally greedy algorithm.

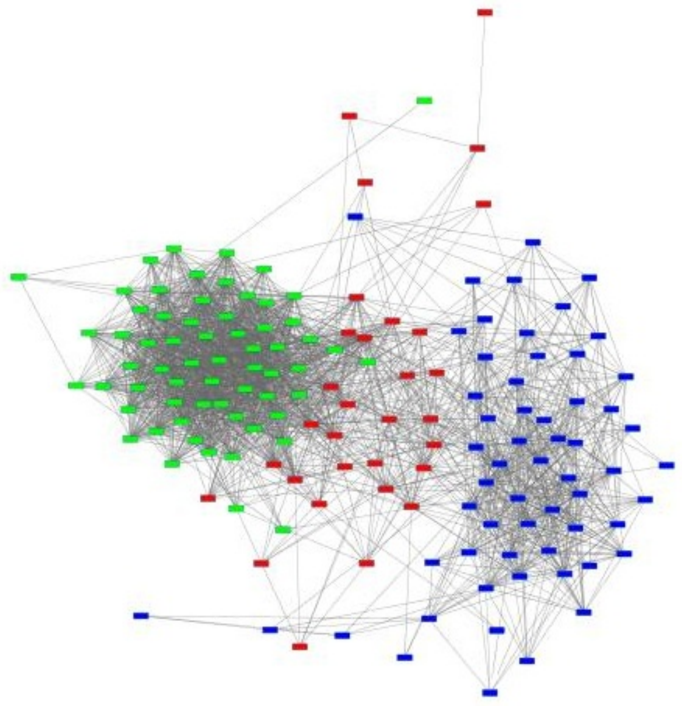

Supplemental Figure 4:

A. Rand similarity index obtained by comparing the consensus partitions of the healthy and IBD control networks (black) and the null model index obtained from permutation testing (gray)

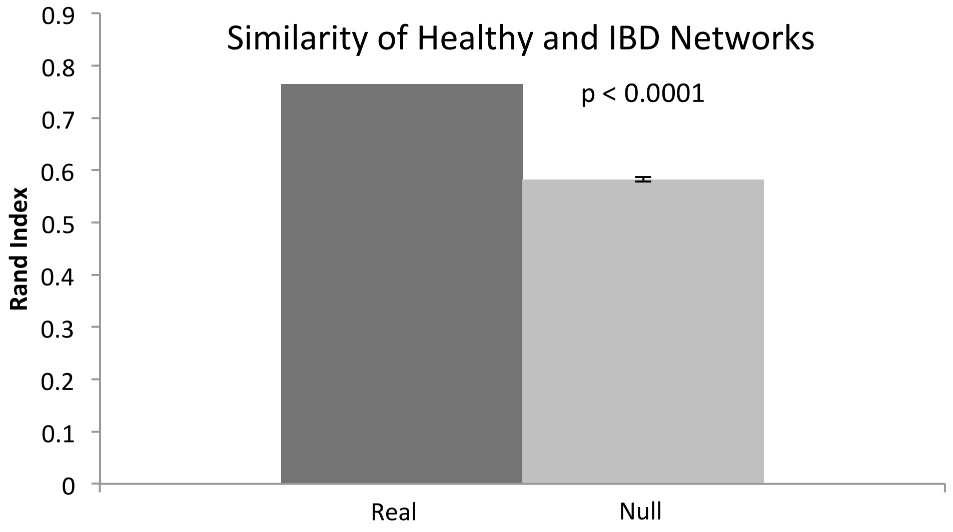

B. Rand similarity index obtained by comparing the consensus partitions of the healthy network (estimated using either the Louvain-like locally greedy algorithm or the edge betweenness-centrality method (black) and the null model index obtained from permutation testing (gray)

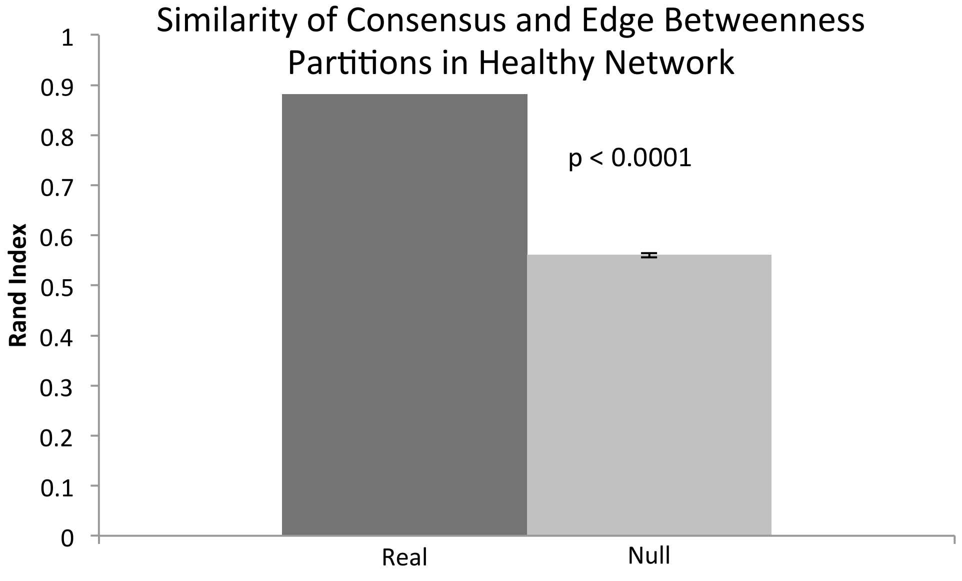

Supplemental Figure 5:

All nodes are color coded by their roles in the healthy scaled distribution (see Figure 4).

A. Plot of absolute (non-scaled) in-module degree *vs*. out-of module-degree in the healthy network.

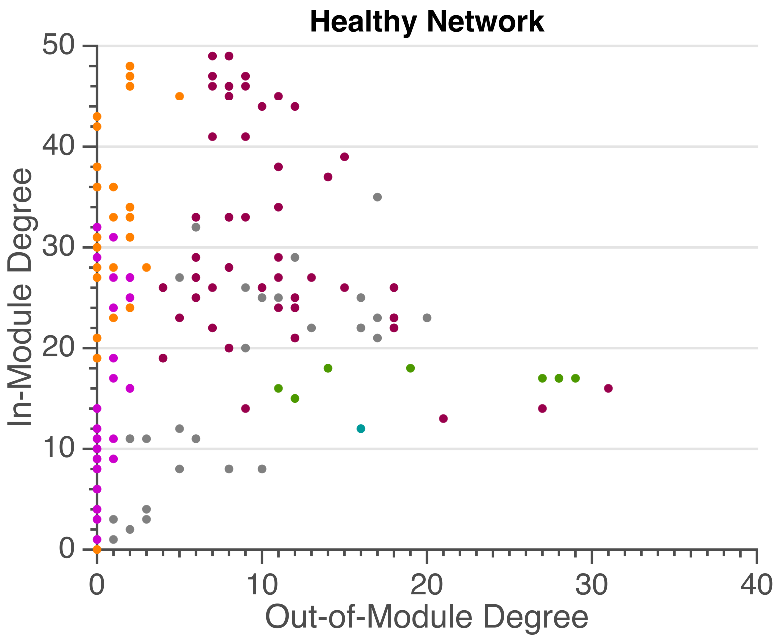

B. Plot of absolute (non-scaled) in-module degree vs. out-of module-degree in the IBD network.

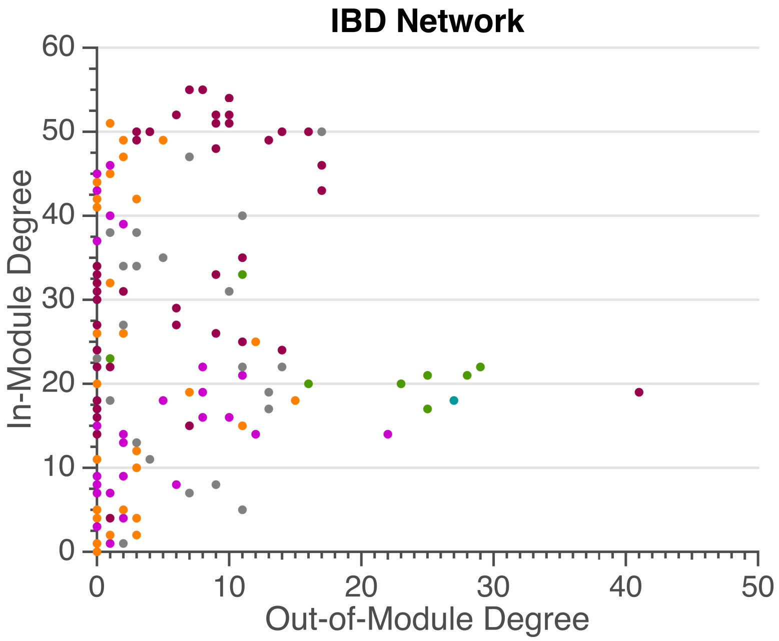

Supplemental Figure 6:

Robustness Analysis

A.

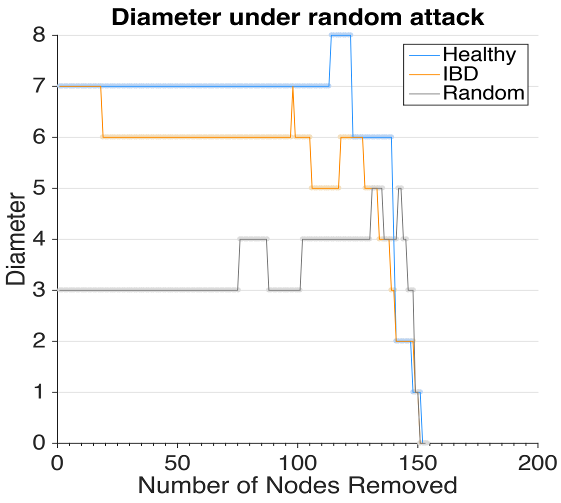

B.

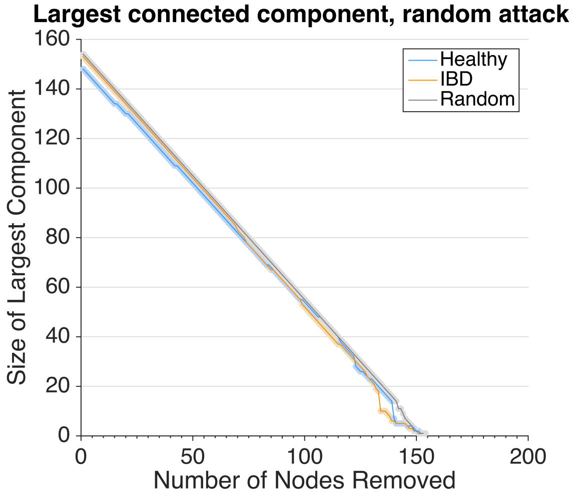

C.

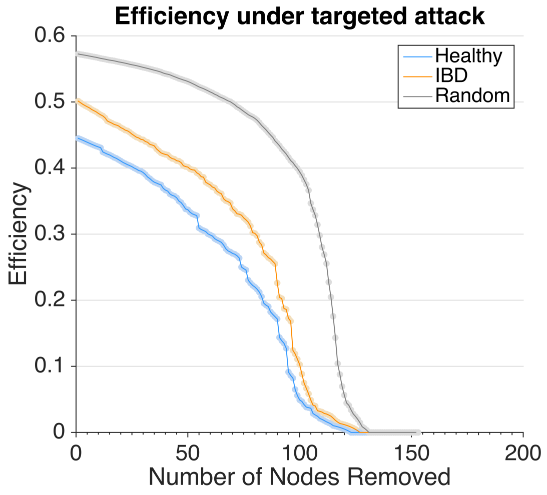

D.

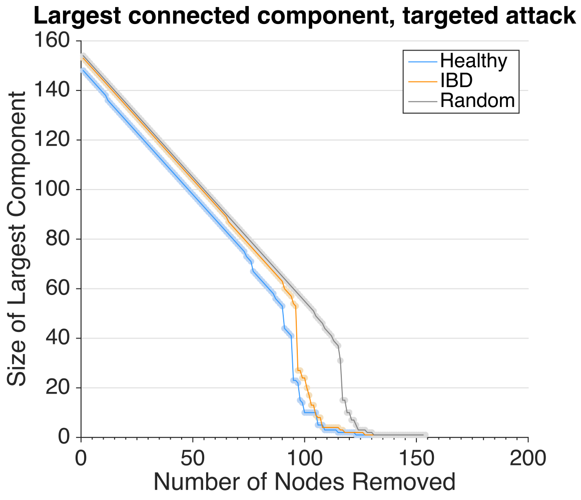

Supplemental Figure 7:

The number of consensus communities detected and the value of the maximum modularity (using the Louvain-like locally greedy algorithm) increase as a smaller fraction of the strongest connections are retained in the binarized adjacency matrix. We chose to use the strongest 20% of connections in the binarized adjacency matrix studied in the main manuscript to limit the effect of noise and reduce computational complexity. Note that the 50th percentile of connection strength corresponds to a Jaccard index of 0.09, implying that connections below this point are unlikely to be functionally significant. Therefore, the threshold chosen ensures the analysis takes place on a representative plateau for number of communities detected. In addition, while the maximum modularity changes with threshold, the modularity of the system is between 0.4 and 0.5 over this plateau. This relatively narrow range indicates that the conclusions drawn regarding the modularity of the system at the threshold chosen are robust.

A.

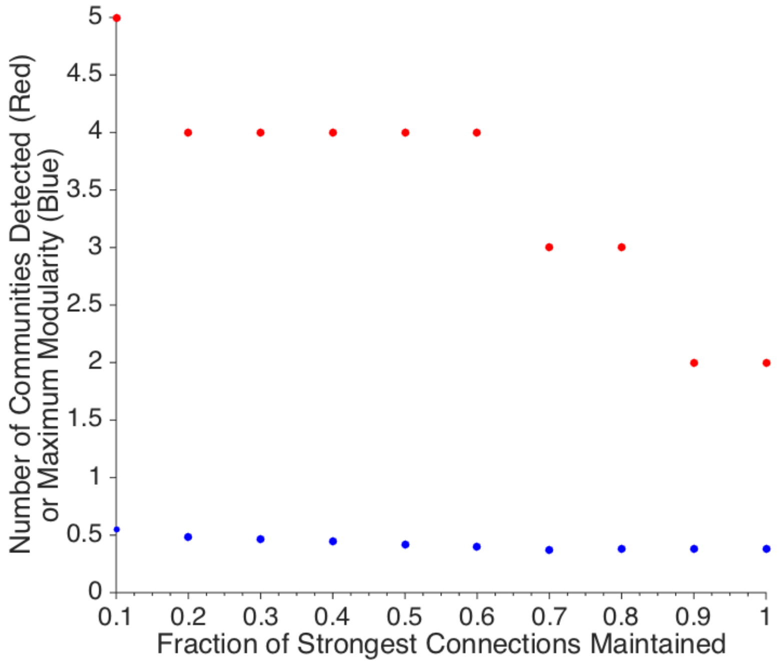

B. The distribution of Jaccard similarity indices that passed this threshold is shown below for each network. Both the absolute threshold and the distribution of edge weights above this threshold are comparable between the two networks.

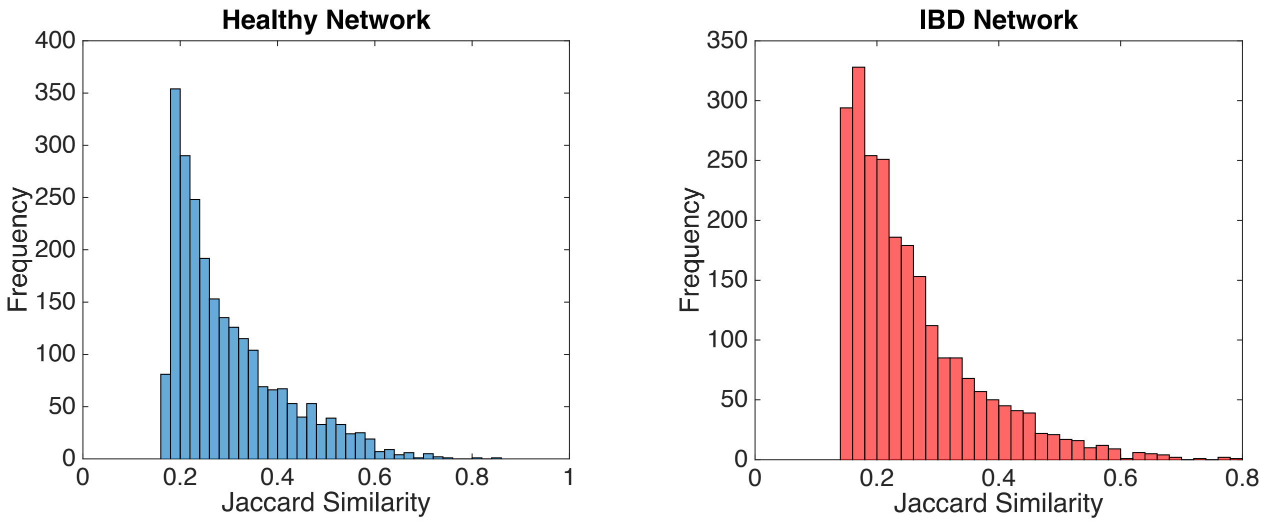

Supplemental Figure 8:

A. Modularity index of the healthy network as determined by the Louvain-like locally greedy algorithm as a function of the structural resolution parameter, **ɣ**.

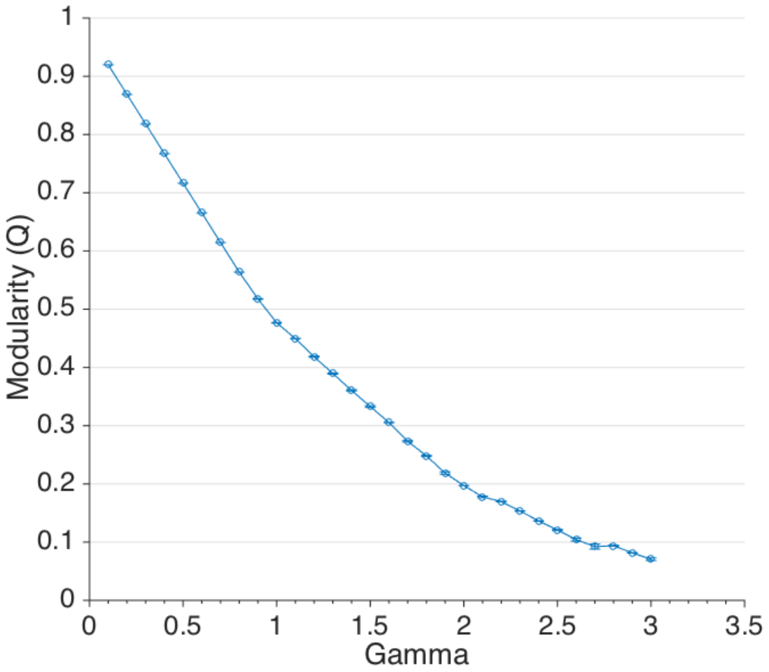

B. Consensus community structure of the healthy network obtained when **ɣ** = 0.5.

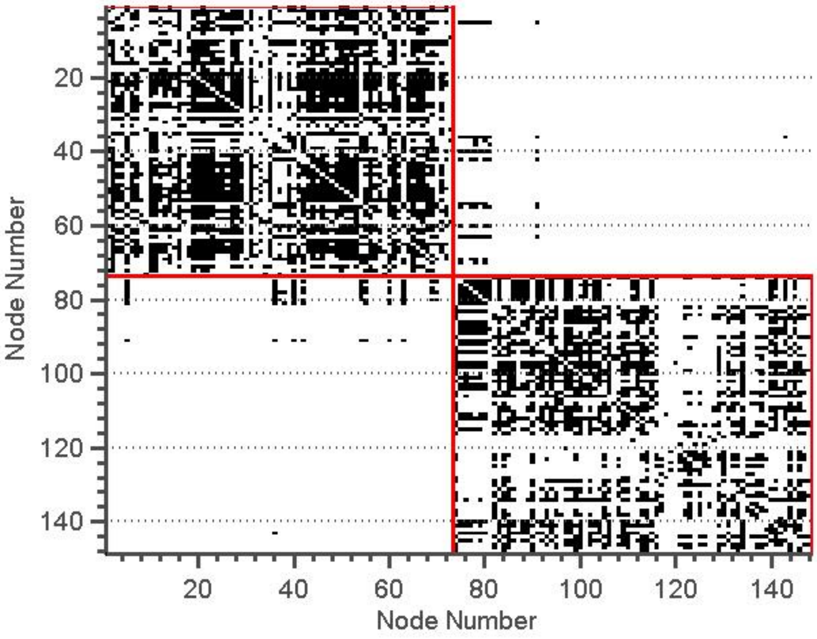

C. Consensus community structure of the healthy network obtained at **γ** = 1.

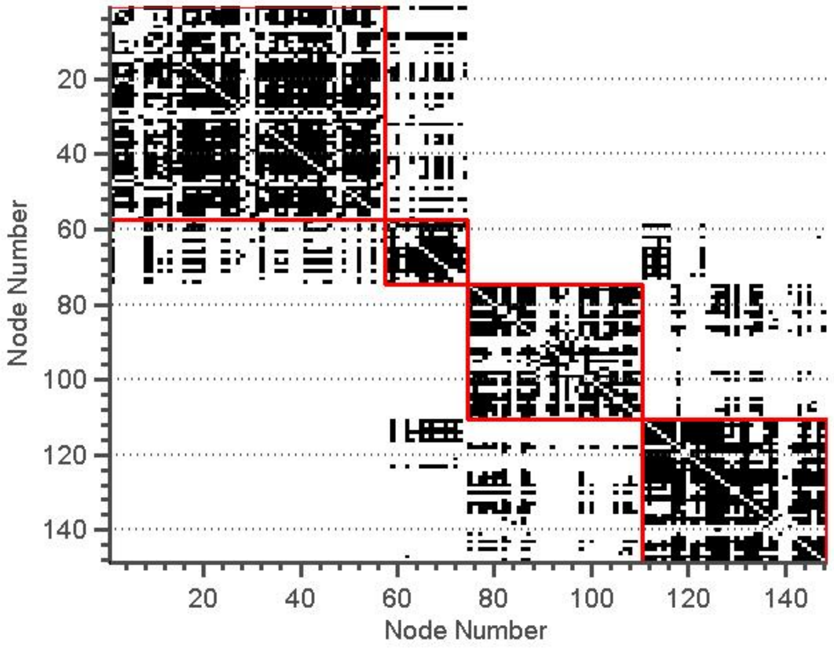

D. Consensus community structure of the healthy network obtained at **γ** = 2.

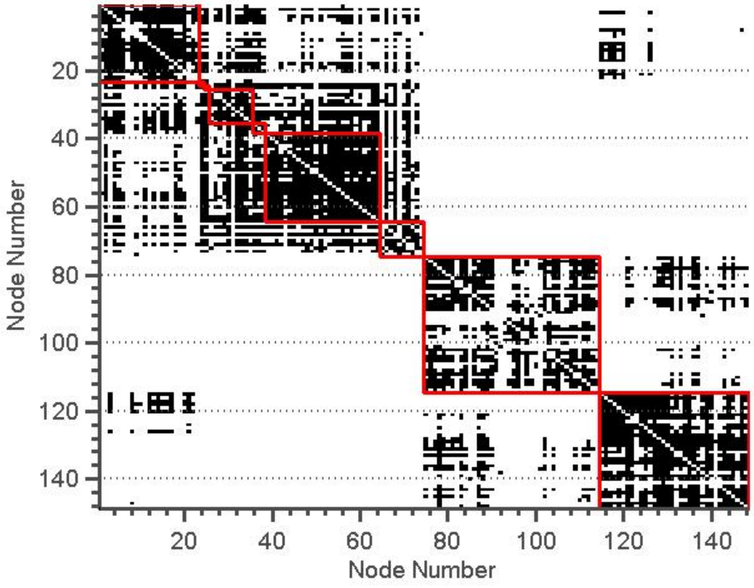

Supplemental Figure 9:

Histogram of degree distribution in Healthy, IBD, Random, and Lattice Networks.

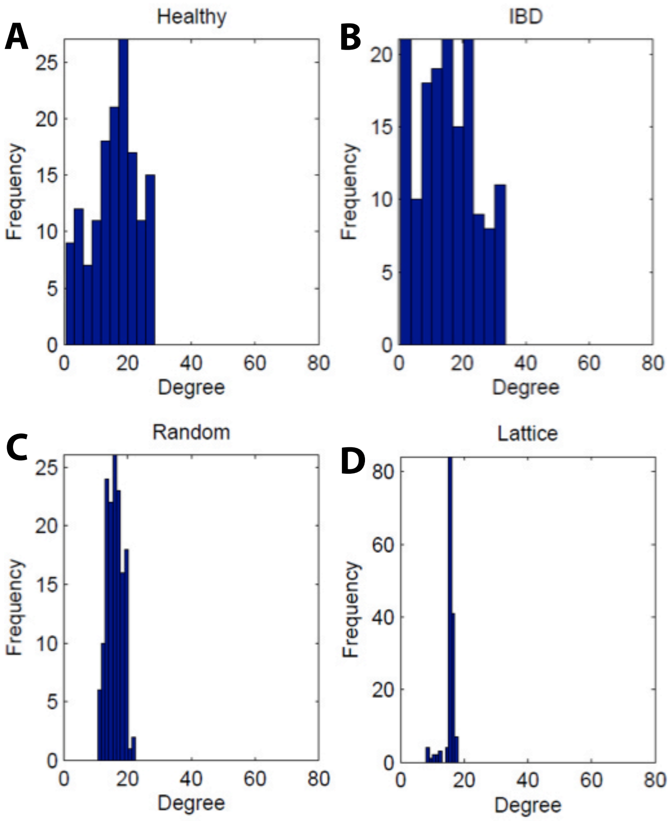

Supplement: Supplementary Information [file srep26087-s1.pdf]
